# Enhancing DNA barcode reference libraries by harvesting terrestrial arthropods at the Smithsonian's National Museum of Natural History

**DOI:** 10.3897/BDJ.11.e100904

**Published:** 2023-04-24

**Authors:** Bernardo F. Santos, Meredith E. Miller, Margarita Miklasevskaja, Jaclyn T. A. McKeown, Niamh E. Redmond, Jonathan A. Coddington, Jessica Bird, Scott E. Miller, Ashton Smith, Seán G. Brady, Matthew L. Buffington, M. Lourdes Chamorro, Torsten Dikow, Michael W. Gates, Paul Goldstein, Alexander Konstantinov, Robert Kula, Nicholas D. Silverson, M. Alma Solis, Stephanie L. deWaard, Suresh Naik, Nadya Nikolova, Mikko Pentinsaari, Sean W. J. Prosser, Jayme E. Sones, Evgeny V. Zakharov, Jeremy R. deWaard

**Affiliations:** 1 National Museum of Natural History, Smithsonian Institution, Washington, United States of America National Museum of Natural History, Smithsonian Institution Washington United States of America; 2 Institut de Systématique, Evolution, Biodiversité (ISYEB), Muséum National d’Histoire naturelle, CNRS, SU, EPHE, UA, Paris, France Institut de Systématique, Evolution, Biodiversité (ISYEB), Muséum National d’Histoire naturelle, CNRS, SU, EPHE, UA Paris France; 3 Centre for Biodiversity Genomics, University of Guelph, Guelph, Canada Centre for Biodiversity Genomics, University of Guelph Guelph Canada; 4 Systematic Entomology Laboratory, Beltsville Agricultural Research Center, Agricultural Research Service, U.S. Department of Agriculture, Washington, United States of America Systematic Entomology Laboratory, Beltsville Agricultural Research Center, Agricultural Research Service, U.S. Department of Agriculture Washington United States of America; 5 Department of Integrative Biology, University of Guelph, Guelph, Canada Department of Integrative Biology, University of Guelph Guelph Canada; 6 School of Environmental Sciences, University of Guelph, Guelph, Canada School of Environmental Sciences, University of Guelph Guelph Canada

**Keywords:** COI, cox1, dark taxa, OTUs, BINs, natural history collection, museum harvesting, National Museum of Natural History, USNM, Centre for Biodiversity Genomics, CBG

## Abstract

The use of DNA barcoding has revolutionised biodiversity science, but its application depends on the existence of comprehensive and reliable reference libraries. For many poorly known taxa, such reference sequences are missing even at higher-level taxonomic scales. We harvested the collections of the Smithsonian’s National Museum of Natural History (USNM) to generate DNA barcoding sequences for genera of terrestrial arthropods previously not recorded in one or more major public sequence databases. Our workflow used a mix of Sanger and Next-Generation Sequencing (NGS) approaches to maximise sequence recovery while ensuring affordable cost. In total, COI sequences were obtained for 5,686 specimens belonging to 3,737 determined species in 3,886 genera and 205 families distributed in 137 countries. Success rates varied widely according to collection data and focal taxon. NGS helped recover sequences of specimens that failed a previous run of Sanger sequencing. Success rates and the optimal balance between Sanger and NGS are the most important drivers to maximise output and minimise cost in future projects. The corresponding sequence and taxonomic data can be accessed through the Barcode of Life Data System, GenBank, the Global Biodiversity Information Facility, the Global Genome Biodiversity Network Data Portal and the NMNH data portal.

## Introduction

The use of DNA barcoding has revolutionised how biodiversity can be surveyed and identified, with applications in fields as broad as biodiversity assessment, invasive species monitoring, agricultural pest control, identification of disease vectors, integrative taxonomy and evolutionary studies (reviewed in Hubert and Hanner (2015)). However, the accuracy of DNA barcoding identifications depends to a large degree on the availability of comprehensive reference libraries, which allow the assignment of scientific names to operational taxonomic units (OTUs), delimited by analysis of barcoding sequences. The construction of reliable reference libraries, often region- or taxon-specific, has received a lot of attention in recent years (e.g. Raupach et al. (2014), Hawlitschek et al. (2015), Morinière et al. (2017), Porco et al. (2018),Weigand et al. (2019), Rimet et al. (2021)). In spite of these advances, assembling reference libraries that can support robust identifications at a broad scale is still challenging for poorly-known taxa, such as many lineages of insects and other terrestrial arthropods with extremely high species number. Identification tools applicable to physical vouchers are often lacking and many taxa (including genera) are known only from a few specimens, often collected decades or even over a century ago (Stork 2018).

In the face of these challenges, one of the most promising avenues for building comprehensive reference libraries is directly harvesting museum specimens that are authoritatively determined (Puillandre et al. 2012, Hebert et al. 2013, Mitchell 2015, Chambers and Hebert 2016, Sire et al. 2019, Rinkert et al. 2021). Major natural history museums often harbour specimens from several thousands of determined species and can support a considerable increase in the availability of reliable entries for barcode reference libraries. The use of such collections, however, is not free of challenges; the sheer scale of collections, diversity of storing and preserving techniques across taxa and the old age of many specimens poses the need to develop optimised, logistic protocols and molecular techniques to amplify and sequence barcoding fragments from often degraded material.

The Smithsonian Institution’s National Museum of Natural History (USNM) comprises the largest natural history collection in the world, with a large portion of its holdings represented by terrestrial invertebrates. For many taxa, the USNM holds the most complete inventory of species of any collection in the world and the vast majority of invertebrate orders have a complete inventory of the holdings at species level. These qualities make it ideally suited to contribute to the general effort of building a global reference library for DNA barcodes, especially for taxa not otherwise represented in repositories such as GenBank (Benson et al. 2012; https://www.ncbi.nlm.nih.gov/genbank/), the Barcode of Life Data System (BOLD; Ratnasingham and Hebert (2007); http://www.boldsystems.org) or Global Genome Biodiversity Network (GGBN; Droege et al. (2014)).

Herein we report results of the project “Barcoding NMNH terrestrial invertebrate genera”, which aims to generate DNA barcoding sequences for genera not previously represented on GenBank, BOLD or GGBN and to initiate the long-term preservation of publicly-accessible genomic DNA extracts and high-resolution images to accompany the physical USNM vouchers. In a companion paper released simultaneously with this one (Levesque-Beaudin et al. 2023), we describe in detail the operational protocol employed. This study aims to focus on the release of the data to provide statistics and metrics for the results of the project to date and to discuss these in the context of the general utility of museum collections in the generation of reference libraries and supporting resources.

## Material and methods

### Specimen Selection and USNM Loan Organisation

In 2018 and 2019, staff from the Centre for Biodiversity Genomics (CBG) completed six visits (46 days total) to the Smithsonian Institution’s National Museum of Natural History, Department of Entomology (USNM). Prior to each visit, a number of target taxa, such as families or superfamilies, were defined, based on number of available specimens, level of curation and physical localisation in the museum. Taxon selection attempted to contemplate most major insect orders, except for Diptera, which were the subject of a pilot project in the development of this methodological workflow (Levesque-Beaudin et al. 2023). Available species inventories for target taxa were compared with the holdings of GenBank and BOLD using a custom application, the GGI Gap Analysis Tool (Global Genome Initiative 2019) to define target genera for sampling. Over the six visits, 8,549 specimens were selected and loaned. Two representatives of different species for each target genus (whenever possible) were selected. Curator specifications, specimen age, collection method, preservation method, number of specimens per genus within the collection and taxonomy were used to determine the appropriate extraction and sequencing protocols for each specimen. Overall, 7,599 specimens were selected for analysis using the CBG’s Sanger-based sequencing protocol (Ivanova et al. 2006) and 950 specimens (mostly specimens older than 60 years and minute specimens of parasitoid wasps) were selected for a protocol involving Next-Generation Sequencing (NGS; see details of the protocol below) (Hebert et al. 2013, Prosser et al. 2016). Of the 7,599 specimens selected for Sanger sequencing, 380 specimens were processed using whole voucher specimens and 7,219 specimens were processed using a tissue sample (leg). Of the 950 specimens selected for NGS, 184 specimens were processed using whole voucher specimens (usually minute Hymenoptera specimens) and 766 specimens were processed using a tissue sample (typically a leg). Specimens were loaned to CBG for processing and sequencing following the 'museum harvesting' protocol developed by Levesque-Beaudin et al. (2023) and detailed below. Specimen data including taxonomy, country of collection, sample ID and specimen cabinet/drawer locations within the USNM collection were recorded by CBG staff at the time of loan organisation.

### Imaging, Digitisation, Tissue Sampling and Sequencing

At the end of each visit, specimens were transferred to CBG for processing. Each specimen was assigned a sample ID, accession number and labelled with a Barcode of Life Data Systems (BOLD) (Ratnasingham and Hebert 2007) specimen label, as well as a unique specimen identifier (USNM ENT) label. Digitisation, imaging and sub-sampling were completed following the protocol outlined in Levesque-Beaudin et al. (2023), following predetermined specifications by USNM museum curators for each taxonomic group. After digitisation, imaging and sub-sampling were complete, data and images were uploaded to BOLD in projects organised by project year and visit (Table A in Suppl. material 1). DNA samples were extracted using the silica-based protocol outlined in Ivanova et al. (2006). PCR amplification followed protocols detailed in Hebert et al. (2013), Prosser et al. (2016) and D'Ercole et al. (2021), targeting overlapping fragments of the cytochrome c oxidase subunit I (COI) gene with two primer sets, (C_LepFolF+MLepR2, 307 bp; and MLepF1+C_LepFolR, 407 bp). PCR protocols and thermal cycler programmes were the same irrespective of sample taxon. All amplicons were visualised on a 2% agarose gel and sequencing amplifications were consolidated into 384-well plates. Bi-directional sequencing was performed on an ABI 3730xl DNA Analyzer (Applied Biosystems, ThermoFisher Scientific). Following sequence editing, sequences were uploaded to BOLD in the appropriate project. Following BOLD upload, DNA extracts were split (20 μl each) with one half stored in the CBG DNA archive and the other sent to the USNM Biorepository. All voucher specimens from the six visits and loans were returned to their original locations within the USNM collection, following the protocol outlined in Levesque-Beaudin et al. (2023).

### NGS pipeline

**Figure 1.**
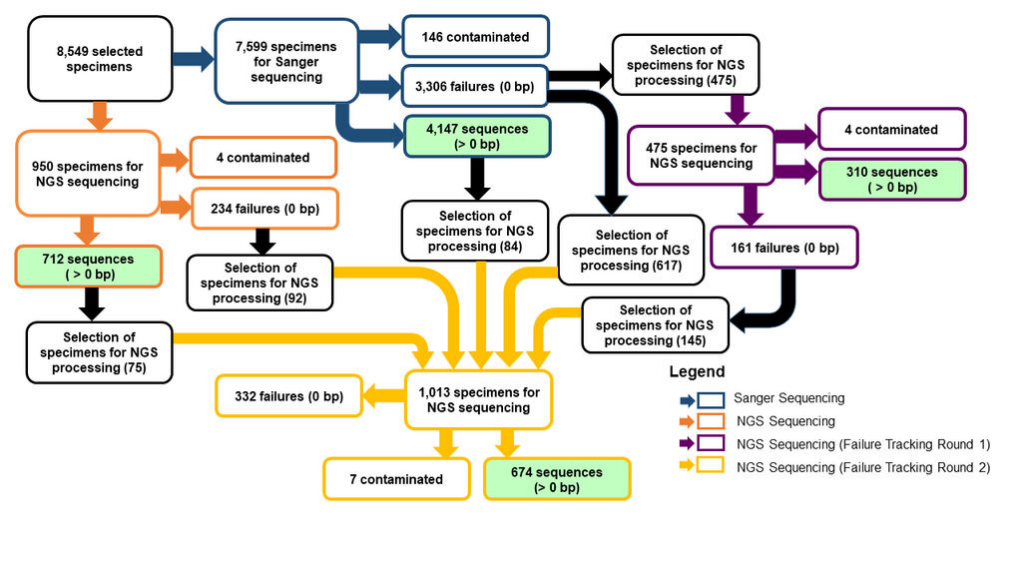
Sanger and NGS Sequencing Flowchart for 8,549 USNM specimens.

From the initial set of specimens, 950 samples were selected for NGS processing; in addition, the NGS pipeline was used for a subset of the specimens that failed to yield sequences using the Sanger protocols. In both cases, the same set of laboratory methods and protocols was adopted. The NGS failure tracking (NGSFT) proceeded as follows: first, a list of genera sampled in Year 1 (Fig. 1) that failed to yield sequences (0 bp) using the Sanger pipeline was compiled and 475 specimens were selected for NGS processing and sequencing (NGSFT Round 1). After this first round was complete, an additional list of genera sampled in Year 1 and Year 2 that failed to yield sequences (0 to 300 bp) using both the Sanger and NGS protocol was compiled, including 143 specimens that failed to yield sequences after the initial round of NGS failure tracking. In NGSFT Round 2, 1013 specimens were selected for NGS processing and sequencing (Fig. 1). Specimen selection was based on genera that would generate the maximum number of unique new GenBank records. All rounds of NGS sequencing followed the same laboratory pipeline, which is based on the multiplexed generation of overlapping short amplicons (150 bp each) (Prosser et al. 2016) that are then sequenced on the PacBio Sequel II.

The complete NGS protocol can be found in Quicke et al. (2020) and D'Ercole et al. (2021) and it is also detailed in the companion paper to this one (Levesque-Beaudin et al. 2023) and can be summarised as follows. Each sample underwent three rounds of PCR amplification. PCR1 aimed at producing a spectrum of COI amplicons from each DNA extract, with three forward primers spanning the barcode region and 5–6 reverse primers (primers outlined in Prosser et al. (2016)). PCR2 aimed at ligating the PacBio “PB1” adapters to the amplicons, providing universal primer binding sites for subsequent fusion of sample-specific unique molecular identifiers (UMIs). PCR3 aimed at adding the UMIs to the amplicons from each specimen so multiple samples could be pooled for sequencing. Following each PCR step, products were purified using a bead-based protocol. The final pools of amplicons were then sequenced with single molecule real time (SMRT) sequencing on the Sequel platform (PacBio; https://www.pacb.com/technology/hifi-sequencing/sequel-system/). The DNA samples used in NGS Failure tracking were stored in the CBG’s DNA Archive.

### Data and Other Resources

All sequences underwent taxonomic validation by matching to existing records using the BOLD ID engine, followed by sequence discordance detection using Neighbour-joining trees of similar taxa (deWaard et al. 2019). Any discordances that indicated contaminated samples resulted in the record being flagged on BOLD and, thus, not a valid DNA barcode. After sequence validation was complete, the successfully sequenced records were added to the BOLD dataset DS-NMNHSEQ, entitled ‘Barcoding NMNH Terrestrial Arthropod Genera’ (http://dx.doi.org/10.5883/DS-NMNHSEQ). All successfully sequenced records (> 200 bp) were made public and submitted to GenBank. USNM voucher information is listed in the “specimen voucher” field of all GenBank records, ensuring the correct linkage with records in the USNM EMu Collection Management System (https://collections.nmnh.si.edu/search/ento). CBG provided the USNM Entomology Data Manager all GenBank Accession numbers, DNA bank data (following the GGBN Data Standard; Droege et al. (2016)) and specimen images which were submitted to the USNM EMu collection management system.

## Data resources

The specimen data, images and sequencing data for all 8,549 specimen records are available on BOLD in the public dataset DS-NMNHALL (http://dx.doi.org/10.5883/DS-NMNHALL) and searchable in the Public Data Portal on BOLD (www.boldsystems.org/index.php/Public_BINSearch) or downloadable by utilising BOLD’s API (www.boldsystems.org/index.php/resources/api).

Specimen records include taxonomy, collection date and location, USNM ENT identifiers, EZID reference numbers (corresponding to EMu-minted records that have globally-unique identifier status), BINs and any additional voucher specimen details. All specimen images are publicly available under the Creative Commons No Rights Reserved (CC0 1.0) licence. All data were submitted and stored in the USNM EMu collection management system and individual records are accessible at https://collections.nmnh.si.edu/search/ento/. Specimen data and DNA storage information were submitted to the Global Genome Biodiversity Network (GGBN) Data Portal (Droege et al. 2014; https://www.ggbn.org/ggbn_portal/search/result?voucherCol=NMNH%2C+Washington).

All sequences have been submitted to GenBank; the dataset can be accessed through NCBI’s BioProject PRJNA81359 (https://www.ncbi.nlm.nih.gov/bioproject/81359). All specimen data have also been uploaded to the Global Biodiversity Information Facility (GBIF; http://www.gbif.org) in the ‘NMNH Extant Specimen Records (USNM, US)’ occurrence dataset (https://doi.org/10.15468/hnhrg3). DNA extracts derived from sequenced specimens are held in the CBG DNA Archive (as specified in deWaard et al. (2019)) and in the NMNH Biorepository (https://naturalhistory.si.edu/research/biorepository).

## Results

A complete list of the 8,549 specimens (including USNM ENT IDs, Process IDs, BOLD IDs, COI sequence length, country of origin, collection date and taxonomy) is provided in Suppl. material 1. Specimens represent 13 orders, 212 families, 4,508 genera and 4,863 identified species collected from 148 countries in all continents. In total, 8,549 label images and 12,096 specimen images (TIF format) were completed by CBG imaging technicians.

Of the 4,508 selected genera, 882 genera were represented by one specimen, 3,421 genera were represented by two specimens, 103 genera were represented by three specimens, 75 genera were represented by four specimens and the remaining 27 genera were represented by five or more specimens. At the time of specimen selection (Table A in Suppl. material 1), 4,415 genera were new to GGBN, 4,117 were new to GenBank and 2,696 were new to BOLD. Initial sequencing, using the Sanger and NGS protocols, resulted in the recovery of 4,706 sequences (> 0 bp), with 4,419 sequences of acceptable length (or 'acceptable bacodes', here defined as > 300 bp), a success rate of 51.69% (Table 1).

NGS-based failure-tracking was conducted in two stages (Fig. 1). In round 1, 475 specimens that failed to gain a sequence (0 bp) using the Sanger method (Table 2) were sequenced using Next-Generation Sequencing, resulting in 310 recovered sequences (> 0 bp). Of the 310 specimens that gained a sequence, 300 were of acceptable barcodes (> 300 bp), resulting in a success rate of 63.2% (Table 2). In round 2 of NGS failure tracking, 1,013 specimens with sequences between 0 and 300 bp were selected, these included 145 specimens that failed to gain a sequence (0 bp) in round 1 of NGS FT (Fig. 1). Round 2 of NGSFT resulted in 674 recovered sequences (> 0 bp). Of the 674 recovered sequences, 501 were acceptable barcodes (> 300 bp), with a success rate of 49.5% (Table 2).

**Figure 2.**
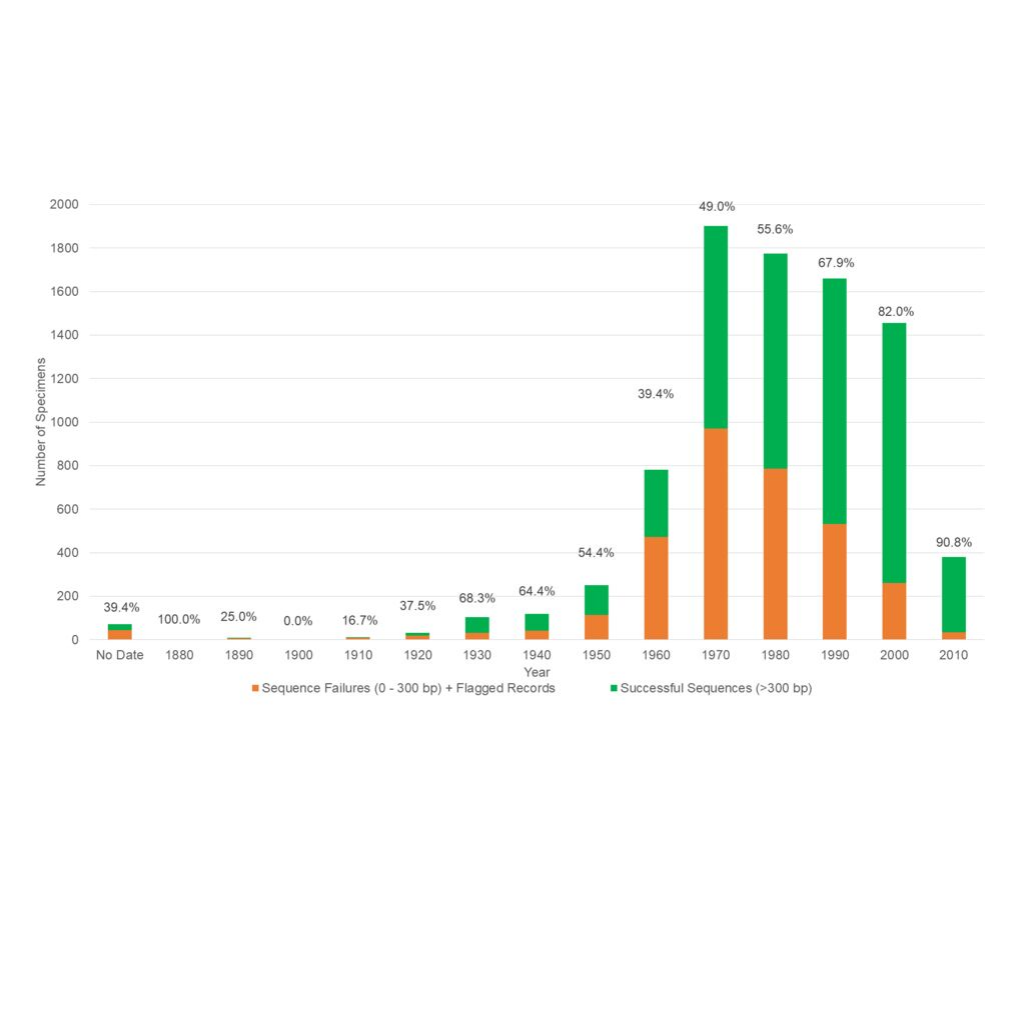
Success length for COI sequencing by specimen collection date (given in percentage values at each bar) for the 8,549 USNM specimens selected in 2018 and 2019. The green bar represents the percentage of specimens collected per decade with recovered sequences (> 300 bp) and orange represents specimens with failed sequences (0 - 299 bp) or flagged sequences.

After NGS-based failure tracking, overall sequence recovery by specimen was 66.5% (5,686 of 8,549 records gained a sequence (> 0 bp) (Table 3). Of the 5,686 records that gained a sequence, 5,220 (61.1%) were acceptable barcodes (> 300 bp) with 3,278 records with sequences 500 bp or greater. Specimen collection dates (by decade) and corresponding sequencing success rates are plotted in Fig. 2.

Of the 4,508 selected genera, 3,886 gained a sequence > 0 bp (86.2%), with 3,638 genera gaining a sequence that was an acceptable barcode (> 300 bp), resulting in a success rate of 80.7% (Table 4). In total, COI sequences (> 0 bp) were obtained for 5,686 specimens belonging to 3,737 species, 3,886 genera and 205 families. The sequences of acceptable barcodes (> 300 bp) constitute 2,437 barcode index numbers (BINs; i.e. a uniquely identified specimen cluster) on BOLD (Ratnasingham and Hebert 2007), with 1,373 unique BINs (56.3%) added to BOLD from this project.

**Figure 3.**
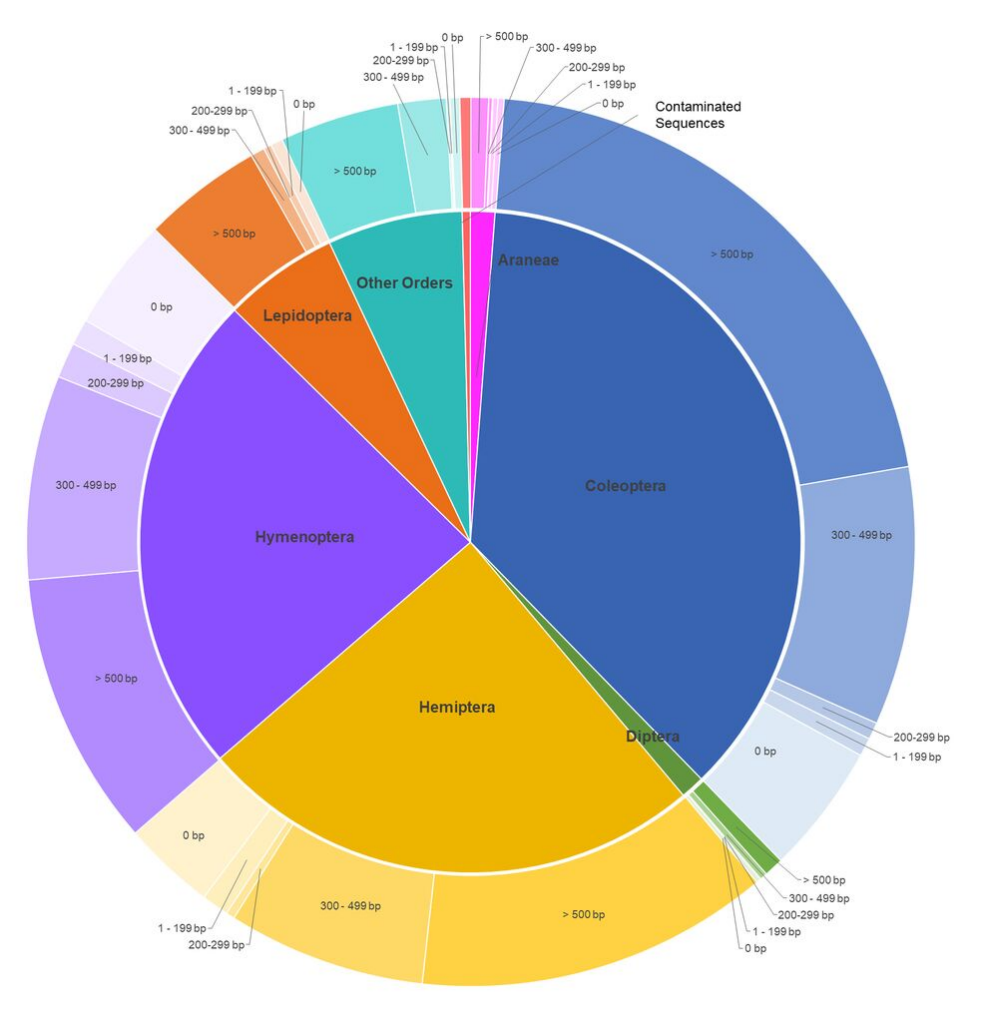
Sequencing results by taxonomic group for 4,508 USNM genera. Inner pie chart shows the proportion of sampled taxa in each taxonomic group and the outer chart shows the distribution of sequencing success within each taxonomic group. **Other Orders**: Mecoptera, Megaloptera, Neuroptera, Odonata, Plecoptera, Raphidioptera and Trichoptera.

Sequence recovery by genera (> 0 bp) for all selected insect orders was between 60.0% and 100.0% (Fig. 3, Table 4). Sequence success by genus for each taxonomic group (> 300 bp) was between 40.0% and 100.0%. Mecoptera had the greatest genus sequencing success (> 0 bp) of all orders with 100.0%, followed by Odonata (97.04%), Neuroptera (94.21%), Lepidoptera (91.02%), Trichoptera (90.91%), Coleoptera (86.71%), Hemiptera (85.93%), Diptera (84.91%), Megaloptera (83.33%), Hymenoptera (82.68%), Araneae (81.48%), Plecoptera (75.0%) and Raphidioptera (60.0%), respectively (Table 4).

Hymenoptera specimens were sequenced using a sample of leg tissue (1,542/2,017 specimens, representing 818 Hymenoptera genera) or using the whole voucher (475/2,017 total specimens, representing 253 Hymenoptera genera), (Table 5). Prior to NGS failure tracking, for specimens sequenced using a leg tissue sample, sequence recovery using the Sanger protocol was 48.40% (652 specimens with sequences > 0 bp), and specimens sequenced with NGS was 65.13% (195 specimens with sequences > 0 bp). For specimens sequenced using the whole voucher, sequence recovery using the Sanger protocol was 47.37% (180 specimens with sequences > 0 bp) and specimens sequenced with NGS was 63.16% (60 specimens with sequences > 0 bp). Prior to NGS failure tracking, genus sequence recovery for leg tissue (using Sanger and NGS protocols combined) was 52.32% (428 of 818 genera > 300 bp) and genus sequence recovery for the whole voucher was 47.43% (120 of 253 genera > 300 bp). After NGS failure tracking, for specimens sequenced using a leg tissue sample, sequence recovery for increased from 50.52% to 64.79% (999 specimens with sequences > 0 bp) and sequence recovery for whole voucher specimens increased from 50.53% to 56.84% (270 specimens with sequences > 0 bp); (Table 6). After NGS failure tracking was complete, genus sequence recovery for leg tissue (using Sanger and NGS protocols combined) increased from 52.32% to 78.73% (644 of 818 genera > 300 bp) and genus sequence recovery for the whole voucher increased from 47.43% to 61.66% (156 of 253 genera > 300 bp).

## Discussion

The persistent scarcity of reliable reference libraries for many poorly-known invertebrate taxa has been a growing concern, reflected in the recent emergence of specific projects and initiatives aimed specifically at such groups, such as “GBOL III: Dark Taxa” by the German Barcode of Life Initiative (Rduch and Peters 2020). Our study intentionally targeted genera that were not represented in existing public databases of barcode sequences, keeping in line with the Global Genome Initiative’s objective of increasing barcode representation along the major branches of the Tree of Life.

Using authoritatively identified material from one of the most prominent natural history collections in the world, we were able to provide novel DNA barcoding data for thousands of genera which had not yet been sequenced and for 3,743 determined species of terrestrial arthropods. This data release represents not only an important advance in the availability of species-level reference barcodes for several taxa, but also has the potential to assist genus-level identifications for groups for which reference sequences are sorely lacking. These results were attained by using a workflow that combines on-site sampling with off-site processing of specimens and DNA extracts (Levesque-Beaudin et al. 2023), with the use of the high-throughput infrastructure at the CBG allowing for the use of the same, standardised workflow and gains of scale in terms of cost and output.

The laboratory protocol used for this study was primarily based on Sanger sequencing, with an NGS pipeline used as an alternative method to recover sequences for very old or small taxa or to specifically target samples that had failed to sequence using the Sanger-based methodology. In our case, this increased overall success, mostly due to the change in amplification strategy (i.e. use of nested PCR targeting smaller fragments; see Hausmann et al. (2009) and Lees et al. (2010) for examples of similar approaches); the NGS sequencing platform probably improves the success rate as well, but the primary advantage of NGS in this pipeline is the decrease in sequencing cost when multiple amplicons per specimens are needed, as well as the reduction in the amount of DNA required for the reactions.

As costs associated with NGS processing continue to decline (National Human Genome Research Institute 2019), we envision a point where our hybrid approach will no longer be cost-effective compared to NGS alone. In strict terms, matching cost levels are achieved when the difference in total cost (C) per specimen (including amplification costs) between NGS and Sanger approaches matches the difference in success rate or efficiency (E) between the two approaches (i.e. when C_Sanger_/E_Sanger_ = C_NGS_/E_NGS_). Monitoring this 'tipping point' is essential for the efficiency of studies aiming to produce reference libraries, but calculating this specific point of inflection is not always straightforward. While the difference in cost per specimen is easily calculable, the difference in efficiency between Sanger and NGS depends on specimen age, size, preservation method and other factors. Many of these variables are often opaque – while specimen age is usually preserved in the labels, means of preservation prior to mounting is usually unknown for each given specimen. In some cases, indirect evidence can be inferred, based on collector name or collection method, as well as specific historic aspects of the material being harvested for DNA. Rimet et al. (2021) list fixative/preservative medium as obligatory metadata for DNA barcoding vouchers of aquatic life, a recommendation that should be followed for terrestrial arthropods as well in vouchering of newly-collected material. As experience accumulates with particular collections, it may become clear that certain collectors used methods that are compatible with Sanger sequencing (Hebert et al. 2013). For example, in moths, different practices include either killing and mounting individual specimens versus holding specimens in humid 'relaxing boxes' for extended periods before mounting, the latter of which is more prone to deteriorate DNA.

In our case, NGS was only attempted for specimens that were either unlikely to be successfully sequenced with Sanger approaches (i.e. very small or old) or as part of failure tracking; hence, our success rates for NGS cannot be used as baseline for overall success if the whole project was conducted under this approach. Overall, our data and those of Levesque-Beaudin et al. (2023) suggest that our NGS pipeline is more appropriate to process decades-old specimens than Sanger-based protocols, meaning that an entirely NGS-based approach may be preferable for studies harvesting largely decades-old material, especially considering the potential evolution of DNA barcoding towards genome skimming (Dodsworth 2015, Coissac et al. 2016, Bohmann et al. 2020). Large-scale studies should consider running pilot projects to investigate differences in efficiency rates amongst different approaches in order to choose an optimal balance.

## Supplementary Material

78C67213-0B27-5776-8536-3DBF6FB2E344 10.3897/BDJ.11.e100904.suppl1 Supplementary material 1 Table S1 Data type Table Brief description Specimen selection visits by CBG staff to the Smithsonian Institution National Museum of Natural History, Department of Entomology (NMNH) and corresponding BOLD project on the Barcode of Life Data Systems (BOLD) (Ratnasingham & Hebert 2007). File: oo_756877.xlsx https://binary.pensoft.net/file/756877 Santos B.F. et al.

## Figures and Tables

**Table 1.** Initial sequencing results by sequencing method for 8,549 USNM specimen records prior to NGS Failure Tracking. 675 genera gained at least one sequence using both the Sanger and NGS protocol during initial sequencing.

**Initial Sequencing Method**	**Total Specimens**	**> 500 bp**	**300–499 bp**	**200–299 bp**	**0–199 bp**	**0 bp**	**Contaminated Sequences**
**Sanger Protocol**	7,599	2,246	1,609	239	53	3,306	146
**NGS Protocol**	950	445	120	63	84	234	4
**TOTAL**	**8,549**	**2,691**	**1,728**	**198**	**89**	**3,693**	**150**
**(% of Total)**		**31.48**%	**20.21**%	**2.32**%	**1.04**%	**43.20**%	**1.75**%

**Table 2.** NGS Failure Tracking sequencing results. A total of 145 specimens failed (0 bp) on the first round of NGS failure tracking and were, therefore, included again in the second round. In total, NGSFT was performed on 1343 specimens.

**Sequencing Method**	**Total Specimens**	**> 500 bp**	**300–499 bp**	**200–299 bp**	**0–199 bp**	**0 bp**	**Contaminated Sequences**
**NGSFT** **(Round 1)**	475	231	69	3	7	161	4
**(% of Total)**		**48.63**%	**14.53**%	**0.63**%	**1.47**%	**33.89**%	**0.84**%
**NGSFT** **(Round 2)**	1,013	356	145	60	113	332	7
**(% of Total)**		**35.10**%	**14.30**%	**5.90**%	**11.20**%	**32.80**%	**0.70**%

**Table 3.** Sequencing results by taxonomic group for 8,549 USNM specimens. **Other Orders**: Mecoptera, Megaloptera, Neuroptera, Odonata, Plecoptera, Raphidioptera and Trichoptera.

**Order**	**Total Specimens**	**> 500 bp**	**300–499 bp**	**200–299 bp**	**1–199 bp**	**0 bp**	**Contaminated Sequences**
**Araneae**	**95**	42	12	1	13	26	1
**Coleoptera**	**3,257**	1284	689	79	41	1095	69
**Diptera**	**103**	44	17	0	1	37	4
**Hemiptera**	**2,042**	776	542	30	58	596	40
**Hymenoptera**	**2,017**	563	493	133	80	736	12
**Lepidoptera**	**454**	281	46	4	13	104	6
Other Orders*	**581**	288	143	11	2	119	18
**Total**	**8,549**	**3,278**	**1,942**	**258**	**208**	**2,713**	**150**
**(% of Total)**		**38.30**%	**22.70**%	**3.00**%	**2.40**%	**31.70**%	**1.80**%

**Table 4.** Sequencing results by taxonomic group for 4,508 USNM genera.

**Order**	**Total Genera**	% **Success (> 300 bp)**	**> 500 bp**	**300–499 bp**	**200–299 bp**	**1–199 bp**	**0 bp**	**Contaminated Sequences**
**Araneae**	**54**	64.5%	29	6	1	8	10	0
**Coleoptera**	**1,655**	83.1%	951	425	29	30	214	6
**Diptera**	**53**	83.0%	32	12	0	1	7	1
**Hemiptera**	**1,123**	80.6%	581	325	14	45	152	6
**Hymenoptera**	**1,068**	73.2%	449	333	58	43	184	1
**Lepidoptera**	**256**	85.9%	197	23	0	13	21	2
**Mecoptera**	**7**	100%	6	1	0	0	0	0
**Megaloptera**	**12**	75.0%	6	3	1	0	2	0
**Neuroptera**	**121**	92.6%	91	21	2	0	6	1
**Odonata**	**135**	96.3%	83	47	1	0	3	1
**Plecoptera**	**8**	62.5%	3	2	0	1	2	0
**Raphidioptera**	**5**	40.0%	2	0	0	1	2	0
**Trichoptera**	**11**	90.9%	5	5	0	0	1	0
**Total**	**4,508**	80.7%	**2,435**	**1,203**	**106**	**142**	**604**	**18**
**(% of Total)**			54.02%	26.69%	2.35%	3.15%	13.40%	0.40%

**Table 5.** Tissue type and sequencing method for 2,017 Hymenoptera specimens prior to NGS Failure tracking.

**Initial Sequencing**	**Total Specimens**	**> 500 bp**	**300 - 499 bp**	**200 - 299 bp**	**1 - 199 bp**	**0 bp**	**Contaminated Records**
**Sanger (leg tissue)**	1,347	260	268	93	31	686	9
**NGS (leg tissue)**	195	68	24	10	25	68	0
**TOTAL**	**1,542**	**328**	**292**	**103**	**56**	**754**	**9**
(% of Total)		**21.27**%	**18.94**%	**6.68**%	**3.63**%	**48.90**%	**0.58**%
**Sanger (Whole Voucher)**	380	57	91	32	0	197	3
**NGS (Whole Voucher)**	95	3	29	20	8	35	0
**TOTAL**	**475**	**60**	**120**	**52**	**8**	**232**	**3**
(% of Total)		**12.63**%	**25.26**%	**10.95**%	**1.68**%	**48.84**%	**0.63**%

**Table 6.** Tissue type and sequencing method for 2,017 Hymenoptera specimens after NGS Failure tracking.

	**Total Specimens**	**> 500 bp**	**300 - 499 bp**	**200 - 299 bp**	**1 - 199 bp**	**0 bp**	**Contaminated Records**
**Leg Tissue**	1,542	487	353	87	72	534	9
(% of Total)		**31.58**%	**22.89**%	**5.64**%	**4.67**%	**34.63**%	**0.58**%
**(Whole Voucher)**	475	76	140	46	8	202	3
(% of Total)		**16.00**%	**29.47**%	**9.68**%	**1.68**%	**42.53**%	**0.63**%
